# Small-angle neutron scattering studies suggest the mechanism of BinAB protein internalization

**DOI:** 10.1107/S2052252519017159

**Published:** 2020-01-25

**Authors:** Mahima Sharma, Vinod K. Aswal, Vinay Kumar, R. Chidambaram

**Affiliations:** a Protein Crystallography Section, Radiation Biology & Health Sciences Division, Bhabha Atomic Research Centre, Mumbai 400 085, India; b Solid State Physics Division, Bhabha Atomic Research Centre, Mumbai 400 085, India; c Homi Bhabha Professor, Bhabha Atomic Research Centre, Mumbai 400 085, India

**Keywords:** SANS, Cqm1 dimer, protein deuteration, Cqm1–BinB complex, contrast matching, BinAB internalization, deuterated BinB

## Abstract

Small-angle neutron scattering (SANS) is one of the most widely used neutron-based approaches to study the solution structure of biological macromolecular systems. The selective deuterium labelling of different protein components of a complex provides a means to probe conformational changes in multiprotein complexes. The *Lysinibacillus sphaericus* mosquito-larvicidal BinAB proteins exert toxicity through interaction with the receptor Cqm1 protein; however, the nature of the complex is not known. Rationally engineered deuterated BinB (dBinB) protein from the *L. sphaericus* ISPC-8 species was synthesized using an *Escherichia coli*-based protein-expression system in M9 medium in D_2_O for ‘contrast-matched’ SANS experiments. SANS data were independently analysed by *ab initio* indirect Fourier transform-based modelling and using crystal structures. These studies confirm the dimeric status of Cqm1 in 100% D_2_O with a longest intramolecular vector (*D*_max_) of ∼94 Å and a radius of gyration (*R*_g_) of ∼31 Å. Notably, BinB binds to Cqm1, forming a heterodimeric complex (*D*_max_ of ∼129 Å and *R*_g_ of ∼40 Å) and alters its oligomeric status from a dimer to a monomer, as confirmed by matched-out Cqm1–dBinB (*D*_max_ of ∼70 Å and *R*_g_ of ∼22 Å). The present study thus provides the first insight into the events involved in the internalization of larvicidal proteins, likely by raft-dependent endocytosis.

## Abstract 2

This study reports a solution structural investigation of the Cqm1–BinB protein complex using ‘contrast-matched’ small-angle neutron scattering (SANS). The SANS data clearly reveal that BinB binds to the receptor Cqm1 and alters its oligomeric status from a dimer to a monomer.

## 1. Introduction   

Small-angle neutron scattering (SANS) is one of the most widely used neutron-based approaches for solution structure studies of biomacromolecular complexes where crystallization proves to be intractable. Selective deuterium labelling of the component proteins combined with contrast variation can be very useful to distinguish between and model different regions of multi-protein complexes (Neylon, 2008 ▸; Dunne *et al.*, 2017 ▸). Protein deuteration can be achieved by three different approaches, including deuteration through expression of the protein in D_2_O-based medium but using an unlabelled (hydrogenous) carbon source, which may yield 60–70% D incorporation with a scattering length density (SLD) close to ∼99% D_2_O (Koruza *et al.*, 2018 ▸; Dunne *et al.*, 2017 ▸).

Binary (BinAB) toxin is responsible for the mosquito-larvicidal properties of *Lysinibacillus sphaericus*. The two component proteins, BinA (the toxic component) and BinB (the receptor-binding component), work synergistically and exert toxicity through interaction with the receptor, Cqm1, in *Culex quinquefasciatus* (Darboux *et al.*, 2001 ▸). Bound apically to the epithelial membrane of the larval midgut cells via a glycosylphosphatidylinositol (GPI) anchor, Cqm1 mediates toxin internalization. Presentation of the receptor on the cell membrane and interaction of the toxin component proteins with the receptor are essential for the larvicidal cytotoxicity. The intracellular toxicity of the BinA component has been proposed to be associated with its glycan affinity (Sharma *et al.*, 2018*a* ▸). However, the mode of toxin internalization has long been debated and remains unclear (Oei *et al.*, 1992 ▸; Opota *et al.*, 2011 ▸; Lekakarn *et al.*, 2015 ▸). The crystal structures of the Cqm1 and BinAB proteins are known (Sharma & Kumar, 2019 ▸; Colletier *et al.*, 2016 ▸); however, the structure of the biological complex is not known. Understanding the details of their interaction may provide clues to the toxin internalization mechanism.

Here, a SANS study of Cqm1–BinB interaction using hydrogenous BinB (hBinB) and deuterated BinB (dBinB) is reported. High-yield expression of dBinB protein using an unlabelled carbon source was achieved and the homogenously purified dBinB protein was characterized for proper folding, thermal stability and its interaction with the receptor protein. The Cqm1 protein was investigated in its solution form and in complex with BinB. The SANS study shows that Cqm1 exists as a dimer in solution and undergoes a change in oligomeric status from a dimer to a monomer upon interaction with BinB. The present study also provides low-resolution details of the complex structure and clues to the mechanism of toxin internalization.

## 2. Materials and methods   

### 2.1. Materials   

Restriction enzymes and Phusion polymerase were obtained from New England Biolabs. The expression vector pET-28a(+) was obtained from Novagen. LB broth and LB agar were procured from HiMedia Laboratories. *Escherichia coli* XL-10 Gold and BL21 Star (DE3) bacterial strains were obtained from Stratagene and Novagen, respectively. Ni–IDA matrix was obtained from GE Healthcare. Isopropyl β-d-1-thiogalactopyranoside (IPTG), kanamycin, phenylmethyl­sulfonyl fluoride (PMSF) and SYPRO Orange dye were obtained from Sigma. All other chemicals were of analytical grade.

### 2.2. Rational engineering of BinB and purification of BinB and Cqm1   

The yield of full-length BinB protein from the recombinant plasmid carrying the *bin*B gene from *L. sphaericus* ISPC-8 was observed to be low. The use of different solubility tags or expression vectors did not improve the yield of soluble and properly folded protein. Overlapping the *L. sphaericus* ISPC-8 BinB protein sequence (GenBank accession ID EU375309.1) with other BinB sequences available in GenBank revealed that the *L. sphaericus* ISPC-8 sequence differs from the other sequences primarily at positions 109 and 274 [His109 and Pro274; Supplementary Fig. S1(*a*)]. These residues occupy surface positions, as revealed from the available BinB crystal structure [PDB entry 5foy; Colletier *et al.*, 2016 ▸; Supplementary Fig. S1(*b*)]. Hence, we restored the invariant Pro109 and Ser274 residues by the overlap-extension method (Section S1.1, supporting information). DNA sequencing confirmed the substitutions.

The ‘rationally’ engineered pET-28a-*bin*B construct was transformed into *E. coli* BL21 Star (DE3) cells for protein expression. BinB protein (with an N-terminal 6×His tag of 18 residues) was purified using immobilized metal ion-affinity chromatography (IMAC) using Ni–IDA matrix (Section S1.2, supporting information).

The receptor Cqm1 (functional form; residues 23–560), lacking the 22 N-terminal signal peptide residues and 20 C-terminal GPI anchor residues, was expressed in a soluble form using *E. coli* BL21 Star (DE3) cells with an N-terminal polyhistidine tag of 22 residues and was purified to homogeneity using Ni–IDA affinity chromatography as described previously (Sharma *et al.*, 2018*b* ▸).

Partially deuterated forms of the Cqm1 and BinB proteins were obtained by buffer exchange [using ultracentrifugal devices; Amicon Ultra, molecular-weight cutoff 10 kDa) of the hydrogenous Cqm1 and BinB proteins into D_2_O-based buffer *C* (25 m*M* HEPES pH 7.5, 25 m*M* NaCl in 100% D_2_O). The concentrations of the purified BinB and Cqm1 proteins in buffer *C* were estimated from their absorbance at 280 nm using extinction coefficients (57 105 and 121 700 *M* ^−1^ cm^−1^, respectively) estimated from the amino-acid sequences by the *ProtParam* tool (https://web.expasy.org/protparam/; Gasteiger *et al.*, 2005 ▸).

### 2.3. Expression and purification of deuterated BinB   

To express dBinB protein, *E. coli* BL21 Star (DE3) cells were adapted from H_2_O-based to D_2_O-based M9+ medium (M9 medium supplemented with a high percentage of hydrogenated glucose as the carbon source; Supplementary Table S1) using a three-step approach (Cai *et al.*, 2016 ▸; Supplementary Fig. S2; Section S2, supporting information). The third-stage 25 ml pre-culture was inoculated into 250 ml M9+/D_2_O medium and allowed to grow at 37°C to an OD_600_ of 0.7. Protein expression was induced with 1 m*M* IPTG at 20°C. The cells were grown further for an extended period of 48 h at 20°C before harvesting. This protocol ensured a high yield of deuterated protein even using conventional laboratory cell-culture equipment. The concentration of the purified dBinB protein (in buffer *C*), purified using Ni–IDA matrix, was estimated from the absorbance at 280 nm using an extinction coefficient (57 105 *M* ^−1^ cm^−1^) estimated from the amino-acid sequence.

### 2.4. Biochemical and biophysical characterization of deuterated BinB   

BinB and dBinB proteins (each at 0.5 mg ml^−1^) were monitored to assess their folding state by intrinsic (tryptophan) fluorescence and from estimation of the melting temperature (*T*_m_). Fluorescence spectra were recorded at 25°C on a JASCO spectrofluorometer (FP-8500) over the wavelength range 290–400 nm after excitation at 280 nm. Each spectrum was obtained by averaging three individual scans. For thermal stability analysis using a Thermofluor shift assay, a protein sample (2 µ*M*) was mixed with 5× SYPRO Orange dye and loaded into 96-well plates (Bio-Rad) sealed with Optical Quality Sealing Tape (Bio-Rad). The plate was heated from 20 to 90°C at a ramp rate of 1.0°C min^−1^ and data were recorded on a CFX96 Real-Time PCR Detection System (Bio-Rad) in FRET mode. Each experiment was performed in triplicate. The melting curves were analysed using the *CFX Manager* software (Bio-Rad) and *T*_m_ was determined from the first derivative of the melting curve.

### 2.5. Native PAGE and DLS analysis of proteins   

Cqm1 and BinB proteins were mixed in a 1:2 molar ratio and maintained at 25°C for 1 h. The formation of the stable complex was adjudged on a 10% native PAGE gel and by dynamic light scattering (DLS) performed on a Zetasizer Nano ZS instrument (Malvern Instruments). For DLS, three sets of measurements were recorded at 25°C for each of the proteins (0.5 mg ml^−1^; Cqm1, BinB and a mixture of the Cqm1 and BinB proteins) and data analysis was performed using the *Zetasizer* software v.7.01 (Malvern Instruments).

### 2.6. SANS data collection and analysis   

SANS measurements were carried out at the SANS-I facility at the Dhruva reactor, Bhabha Atomic Research Centre, Mumbai, India (Aswal & Goyal, 2000 ▸). Neutrons with a wavelength (λ) of 5.2 Å and a wavelength spread Δλ/λ of 15% were selected using the neutron velocity selector. Scattered neutrons were detected using a 1 m long ^3^He position-sensitive detector. The data were collected in a *q* range (*q* = 4πsinθ/λ, where 2θ is the scattering angle) from 0.015 to 0.26 Å^−1^. Samples were held in quartz cells of 5 mm thickness and the temperature was maintained at 25°C. All data were corrected for solvent and background, and normalized to cross-sectional units using a standard procedure. Owing to high incoherent scattering at high *q*, the data sets were truncated for *q* > 0.2 Å^−1^. The concentrations of the different proteins used in SANS data collection were 3.3 mg ml^−1^ for Cqm1, 3.3 mg ml^−1^ for hBinB, 5 mg ml^−1^ for the Cqm1–BinB complexes and 6 mg ml^−1^ for dBinB. The reduced SANS intensity was normalized to a protein concentration of 1 mg ml^−1^ to estimate *I*(0) (the intensity at *q* = 0).

The SANS data were analysed and modelled using software tools available in *ATSAS* 2.8 (Franke *et al.*, 2017 ▸) following two independent approaches: *ab initio* modelling by the indirect Fourier transformation (IFT) method using *DATGNOM* (Petoukhov *et al.*, 2007 ▸) and using available structural information (theoretical) by the *CRYSON* module of *ATSAS* 2.8 (Svergun *et al.*, 1995 ▸). The radius of gyration (*R*_g_) and absolute intensity *I*(0) (at *q* = 0) values were estimated from the pair-distance distribution [*P*(*r*)]. The molecular weights (MW) of the proteins were estimated by MW = *I*(0) × (*N*_A_ *d*_p_^2^/Δρ^2^), where *N*_A_ is Avogadro’s number, the average protein density (*d*_p_) is 1.36 g cm^−3^ and the average excess scattering length density of proteins, Δρ, is 2.8 × 10^10^ cm^−2^. *Ab initio* modelling of the shape was achieved with the online *DAMMIN* module (Svergun, 1999 ▸) using *P*(*r*) values and the automatic algorithm available on the *ATSAS* online server (https://www.embl-hamburg.de/biosaxs/atsas-online/; Franke *et al.*, 2017 ▸). The experimental and theoretical Kratky plots were also calculated using *Origin* and are given in Supplementary Fig. S6.

The agreement between observed scattering and transformed data were assessed using the reduced χ^2^ values defined as
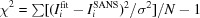
where the summation is over all observed experimental data points *i*, *I*_*i*_^fit^ and *I*_*i*_^SANS^ are the fitted and observed intensities for the *i*th observation, σ_*i*_ is the experimental error and *N* is the number of data points in the analysed *q* range.

The atomic coordinates of Cqm1 (residues 7–537) and BinB (residues 28–446) monomers were extracted from Protein Data Bank (PDB) entries 6k5p (Sharma & Kumar, 2019 ▸) and 5foy (Colletier *et al.*, 2016 ▸), respectively. The template-based CA-CA-guided docking method was used to model the Cqm1–BinB complex structure using the *HADDOCK* web server (Xue *et al.*, 2017 ▸) and the interfacial residues of Cqm1 (Ser109, Gly139-Gly140 and Ala292; Ferreira *et al.*, 2014 ▸) and of the BinB protein (Phe41-Tyr42-Asn43; Singkhamanan *et al.*, 2013 ▸).

The atomic structures were fitted into *ab initio* shape models generated with *DAMMIN* using the *SUPALM* and *SUPCOMB* modules of *ATSAS*.

## 3. Results and discussion   

Mosquito-larvicidal binary toxin (BinAB) is highly active against *Culex* and *Anopheles* mosquitoes but is refractory to *Aedes aegypti*. The incompetence of the BinAB toxin against *Aedes* may be due to the inability of the toxin to be internalized across the cell membrane (Lekakarn *et al.*, 2015 ▸). Methods are required to understand the interaction of the BinAB components with the receptor protein in order to understand the mechanism underlying toxin internalization. BinB interacts with Cqm1 in solution with high affinity (*K*_d_ of ∼10 n*M*; Sharma *et al.*, 2018*b* ▸). In this study, we investigated the oligomeric state of Cqm1 and its complex with the BinB protein using hydrogenated and deuterated BinB by ‘contrast-matched’ SANS. All SANS experiments were carried out in 100% D_2_O. The SANS data were analysed as suggested in the recommendations of the Small-Angle Scattering Validation Task Force (Trewhella *et al.*, 2017 ▸).

### 3.1. Characteristics of deuterated BinB   

**Figure 1:**
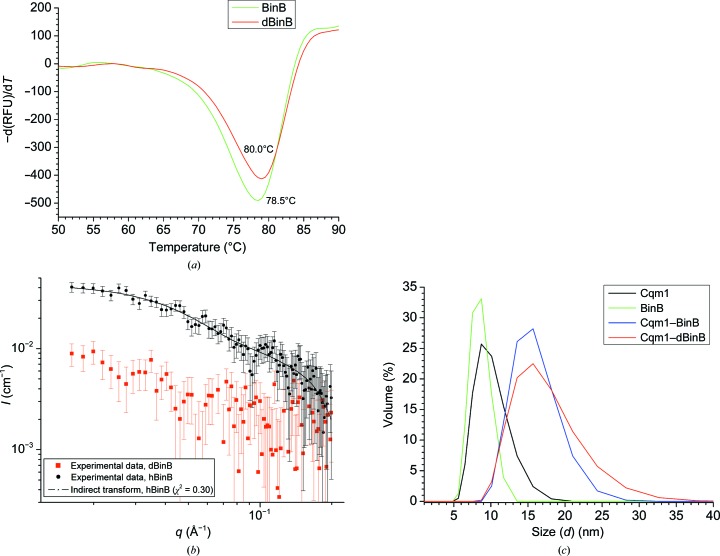
(*a*) Thermal stability analysis of hBinB and dBinB using a Thermofluor shift assay. The dBinB tertiary structure displays a similar thermal stability to its hydrogenous counterpart. (*b*) Experimental SANS data for hBinB (black points) and dBinB (red points) proteins in 100% D_2_O and an *ab initio* IFT fit (black line) for the hBinB SANS data. (*c*) DLS measurements of Cqm1–BinB complexes. The formation of a stable heteromeric complex is evident from the increased hydrodynamic diameter compared with the individual Cqm1 and BinB proteins.

A rationally engineered pET-28a(+)-*bin*B construct was used for expression of the hBinB and dBinB proteins. The three-step approach to adapt *E. coli* cells from H_2_O-based to D_2_O-based culture medium proved to be successful for large-scale purification of the dBinB protein (Supplementary Fig. S2). A high yield of dBinB protein (15 mg dBinB compared with 30 mg hBinB) could be achieved with a cell density of ∼2 (OD_600_) from 1 l *E. coli* culture medium (Supplementary Fig. S3). Notably, an adaptive protocol and a longer induction time (48 h) seem to be critical factors for a higher protein yield. The success of protein deuteration is reflected by its scattering length density (ρ_p_) reaching close to ρ_s_ [that of D_2_O; Fig. 1 ▸(*b*)]. The extent of deuteration for dBinB was estimated from contrast [(ρ_p_ − ρ_s_)^2^] values to be ∼77% (Supplementary Table S2), compared with Cqm1 and BinB deuterated partially through buffer exchange (20% and 16%, respectively). Purified dBinB exhibits proper folding with a λ^em^_max_ of 327 nm [Supplementary Fig. S4(*a*)] and its tertiary structure displays a thermal stability similar to that of the hBinB protein, with a *T*_m_ value of ∼80°C [Fig. 1 ▸(*a*) and Supplementary Fig. S4(*b*)].

### 3.2. SANS modelling   

The SANS curves decrease monotonically over the scattering-vector range 0.016–0.2 Å^−1^. Two independent approaches were employed to achieve fitting to the experimental SANS data: IFT-based *ab initio* modelling and fitting theoretical scattering curves calculated directly from the atomic structures available in the PDB or from docking solutions.

**Figure 2:**
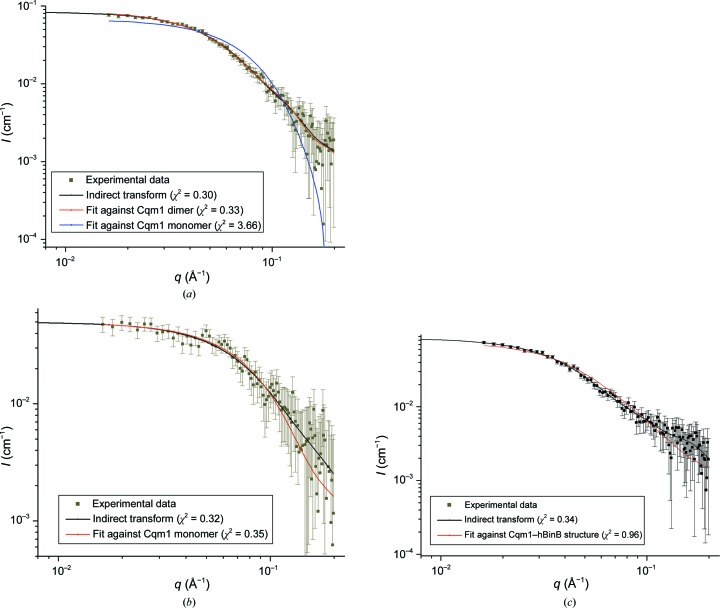
Experimental SANS data for (*a*) Cqm1 protein (black points) in 100% D_2_O, the fit against the X-ray crystal structure of the Cqm1 dimer (red line) and the fit against the crystal structure of the Cqm1 monomer (blue line), (*b*) the Cqm1–dBinB complex (black points) and the resulting fit against the crystal structure of the Cqm1 monomer (red) and (*c*) the Cqm1–hBinB complex in 100% D_2_O buffer (black points) and the resulting fit against the modelled structure of the Cqm1–BinB complex monomer (red). The *ab initio* fit from the IFT method is shown as a black line.

**Figure 3:**
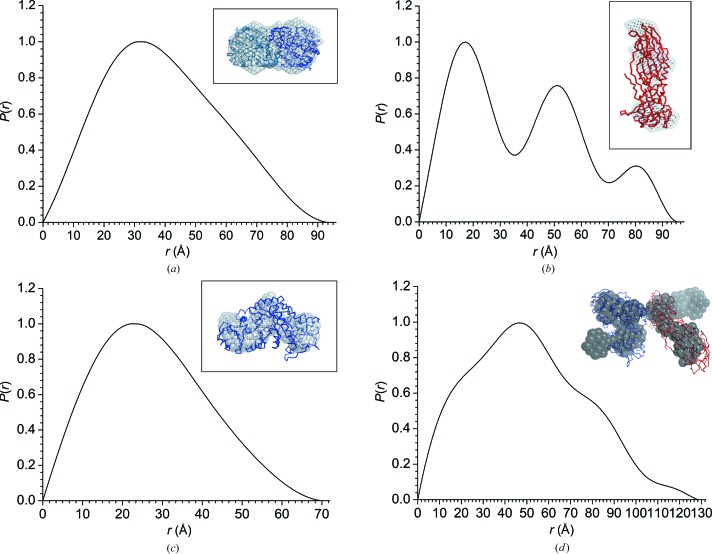
Pair-distance distribution functions [*P*(*r*)] for the experimental (*a*) Cqm1, (*b*) BinB, (*c*) Cqm1–dBinB and (*d*) Cqm1–hBinB data in solution. The *ab initio* shape models generated with *DAMMIN* (grey beads) overlaid with (*a*) the crystal structure of the Cqm1 dimer (blue ribbon), (*b*) the crystal structure of the BinB monomer (red ribbon), (*c*) the Cqm1 monomer (blue ribbon) and (*d*) the derived Cqm1–BinB structure (red ribbon, BinB; blue ribbon, Cqm1 monomer) are shown in the insets. A *P*2 symmetry constraint was used during *DAMMIN* runs for Cqm1 analysis.

The scattering curves obtained by IFT and from a dimeric structure of Cqm1 (Supplementary Fig. S7) match the experimental SANS data, with χ^2^ values close to 0.3 [Fig. 2 ▸(*a*)]. The *R*_g_ (∼31 Å) and *D*_max_ (∼94 Å) obtained by the IFT method with a smooth *P*(*r*) function closely match the theoretical estimates obtained from the dimer structure [Table 1 ▸; Fig. 3 ▸(*a*)]. The molecular weight estimated from the absolute *I*(0) (∼120 kDa) is within 10% of the value deduced from the amino-acid sequences (Table 1 ▸) and a dimer of Cqm1 fits well into the *ab initio* shape model generated with *DAMMIN* [Fig. 3 ▸(*a*)]. In contrast, the theoretical curve for a Cqm1 monomer does not match the experimental SANS data (χ^2^ = 3.6) [Fig. 2 ▸(*a*)]. The dimeric status of Cqm1 in solution also matches the radius of hydration (*R*_h_ = ∼42 Å) observed in dynamic light-scattering experiments [Fig. 1 ▸(*c*)] and the elution profile of the protein from the size-exclusion chromatography column (Sharma *et al.*,, 2018*b* ▸).

Likewise, the BinB monomer matches the SANS and DLS experimental data [Figs. 1 ▸(*b*) and 1 ▸(*c*), Table 1 ▸]. The three oscillations observed in the *P*(*r*) function and the extra bead density observed in the *ab initio* dummy *DAMMIN* model [Fig. 3 ▸(*b*)] can be rationalized owing to the presence of a third domain in BinB constituted of residues 1–45, for which atomic coordinates are not available. In comparison to BinB, the deuterated protein did not result in a significant scattering signal in D_2_O solvent [Fig. 1 ▸(*b*)]. It was thus taken that scattering owing to dBinB was matched out in 100% D_2_O with 77% deuteration.

Single peaks corresponding to *R*_h_ values of about 80 Å in the DLS profiles of Cqm1 mixed with the hBinB and dBinB proteins suggest the formation of stable Cqm1–hBinB and Cqm1–dBinB complexes [Fig. 1 ▸(*c*)], which is also confirmed by the presence of a single protein band, albeit with retarded mobility, on 10% native PAGE (Supplementary Fig. S5). However, analysis of experimental SANS data for the Cqm1–dBinB complex revealed the presence of a Cqm1 monomer in solution [Fig. 2 ▸(*b*)] with the *ab initio* and theoretical curves fitting very well, with χ^2^ values of <0.5 [Figs. 2 ▸(*b*) and 3 ▸(*c*), Table 1 ▸]. A near ‘match-out’ of dBinB against 100% D_2_O used in the SANS experiments explains the data.

Fitting of SANS data for the Cqm1–hBinB complex with *ab initio* IFT modelling indicated *R*_g_ and *D*_max_ values of ∼40 and ∼129 Å, respectively [Table 1 ▸; Figs. 2 ▸(*c*) and 3 ▸(*d*)]. The molecular weight of the complex was estimated to be ∼121 kDa (compared with the molecular weight of 118 kDa expected from the sequence). These data suggested formation of the Cqm1–BinB complex with a 1:1 stoichiometry. Two shoulder peaks, in addition to the main peak at ∼47 Å, in the pair-distance distribution function may suggest the domain structure of the complex. Attempts to match structural models generated by docking analysis using given interaction constraints provided clues to the placement of BinB in the *ab initio* shape model. However, the Cqm1 fit did not seem to be good. Attempts were made to obscure the BinB fitted region and fit the Cqm1 monomer, as observed in the ‘matched-out’ Cqm1–dBinB SANS data, to the remainder of the *ab initio* shape model [Fig. 3 ▸(*d*)]. This results in a low-resolution structural model of the complex, which matches the experimental data with a χ^2^ value of 0.96.

The crystal structure of the Cqm1 protein suggests a weak dimer with a solvation free-energy gain of ∼4.4 kcal mol^−1^ (Sharma & Kumar, 2019 ▸; Supplementary Fig. S7). Each of the monomers is localized on the lipid rafts via a GPI anchor. It can be speculated that the separation of weakly held Cqm1 monomers in the presence of BinB might disrupt the lipid rafts, resulting in raft-dependent endocytosis. A similar mechanism has been suggested for some viral particles, in which lipid-raft disruption enhanced the release of viral particles with compromized infectivity owing to the leakage of essential viral proteins (Barman & Nayak, 2007 ▸).

## 4. Conclusion   

In the present study, we report the first solution structures of the BinAB toxin receptor Cqm1 and of its complex with the BinB protein. Cqm1 exists as a dimer in both H_2_O-based and D_2_O-based buffers. ‘Contrast-matched’ SANS using deuterated BinB and hydrogenous BinB revealed a change in the oligomeric state of the receptor protein from a dimer to a monomer, and provides the first solution model of the Cqm1–BinB complex.

## 5. SASBDB accession codes   

The SANS data have been submitted to the Small Angle Scattering Biological Data Bank (SASBDB; http://www.sasbdb.org; Valentini *et al.*, 2015 ▸) with accession codes SASDF87 (receptor Cqm1 protein), SASDF97 (complex of Cqm1 and dBinB proteins) and SASDFA7 (receptor binding BinB protein).

## 6. Related literature   

The following references are cited in the supporting information for this article: Goujon *et al.* (2010 ▸), Pettersen *et al.* (2004 ▸) and Robert & Gouet (2014 ▸).

## Supplementary Material

SASBDB reference: mosquito-larvicidal binary (BinAB) toxin receptor Cqm1 protein in 100% D_2_O, SASDF87

SASBDB reference: complex of binary toxin receptor (Cqm1) with deuterated BinB component protein in 100% D_2_O, SASDF97

SASBDB reference: receptor binding BinB protein of mosquito-larvicidal binary toxin in 100% D_2_O, SASDFA7

Supplementary Methods, Supplementary Tables and Supplementary Figures. DOI: 10.1107/S2052252519017159/ti5015sup1.pdf

## Figures and Tables

**Table 1:** Values for molecular weight (MW), radius of gyration (*R*_g_) and maximal distance (*D*_max_) MW is as calculated from the SANS data and calculated from the amino-acid sequence. *D*_max_ and *R*_g_ are as determined from the SANS data and from the crystal structure. MW_SANS_, *R*_g,SANS_ and *I*(0) were estimated from the pair-distance distribution. MW_SANS_ was estimated from the mean value of *I*(0). MW_SEQ_ was estimated from the amino-acid sequence. *R*_g,STR_ was estimated from the atomic coordinates. As the coordinates of the N-terminal 45 residues are not available in the atomic structure of BinB, the *R*_g,STR_ values for the protein/complex can be expected to be lower estimates.

	Cqm1 (dimer)	BinB	Cqm1–hBinB complex	Cqm1–dBinB complex†
*I*(0)	0.0850 ± 0.0013	0.04353 ± 0.0024	0.08498 ± 0.0024	0.04963 ± 0.0022
MW_SANS_ (kDa)	120	61.9	121	70.6
MW_SEQ_ (kDa)	129.2	53.6	118.2	64.6
*R*_g,SANS_ (Å)	30.8 ± 0.63	31.6 ± 2.8	40.2 ± 1.74	21.8 ± 1.21
*R*_g,STR_ (Å)	28.3	28.2	38.1	21.3
*D*_max,SANS_ (Å)	93.5	95	128.5	69.5

† In the complex of Cqm1 and deuterated BinB, the scattering length density of BinB nearly matches that of solvent D_2_O and its contribution to the scattering is expected to be eliminated. Thus, values for the Cqm1 monomer were used to estimate MW_SEQ_ and *R*_g,STR_, which closely match the values for MW_SANS_, *R*_g,SANS_ and *D*_max,SANS_ obtained from *ab initio* IFT modelling of SANS data without *a priori* knowledge of atomic structures.
