# Supplementary material for: Small-angle neutron scattering studies suggest the mechanism of BinAB protein internalization
Source: IUCrJ. 2020 Jan 25;7(Pt 2):166–72. doi: 10.1107/S2052252519017159 (PMC7055391; doi:10.1107/S2052252519017159)
Supplement: Supplementary file 1 [file m-07-00166-sup1.pdf]

IUCrJ

Volume 7 (2020)

Supporting information for article:

Small-angle neutron scattering studies suggest the mechanism of BinAB protein internalization

Mahima Sharma, Vinod K. Aswal, Vinay Kumar and R. Chidambaram

S1. Rational engineering of BinB protein for enhancing protein solubility and yield

S1.1. Introduction of point mutations in the *p*ET28a(+)-*bin*B construct using overlap-extension PCR

The recombinant *p*ET28a(+)-*bin*B construct encoding full length BinB protein (ISPC-8) was used as a construct to introduce two point mutations in the *bin*B gene by overlap extension PCR (OE-PCR) translating to two substitutions in BinB protein- His109Pro and Pro274Ser.

OE-PCR based mutagenesis was performed in a two-step PCR procedure. In the first step, two double stranded DNA fragments with overlapping ends carrying the point mutation for His109Pro substitution were generated through PCR using primers 5'-CCGGCGTAGAGGATCGAGAT-3' and 5'-GTTATTATCCAAATAAGTAGGTGCTTCATCAAAAAC-3' (His109Pro product 1) and primers, 5'-GTTTTTGATGAAGCACCTACTTATTTGGATAATAAC-3' and 5'-TAGTTATTGCTCAGCGGTGG-3' (His109Pro product 2). The expected fragments as observed from gel were excised and purified using gel purification kit (Qiagen). In the second step, His109Pro products 1 and 2 were mixed in a 1: 1 ratio and amplified in a modified PCR reaction consisting of 10 cycles in the absence of any primers to produce full length double stranded DNA. Following 10 cycles, extreme end primers, 5'-CCGGCGTAGAGGATCGAGAT-3' and 5'-TAGTTATTGCTCAGCGGTGG-3' were added and the PCR reaction was continued for 25 more cycles to amplify the full length *bin*B gene with point mutation for His109Pro substitution. The *bin*B gene after this stage was used as template for introducing second point mutation for P274S substitution. Primers sets, 5'-ATATACATATGTGCGATTCAAAAGACAATTCTGG-3' and 5'-GCAGGTATAATTTGTGACCATAATTGATGCCAG-3', and 5'-CTGGCATCAATTATGGTCACAAATTATACCTGC and 5'-TATAGGATCCTCATTACTGGTTAATTTTAGG-3' were used to amplify the two PCR fragments with overlapping ends carrying the point mutation for Pro274Ser substitution. In the second step, the PCR products were mixed in a 1: 1 ratio and amplified in a modified PCR reaction consisting of 10 cycles in the absence of any primers to produce full length double stranded mutagenic DNA. Following 10 cycles, *bin*B gene specific primers with NdeI and BamHI restriction sites, 5'-ATATACATATGTGCGATTCAAAAGACAATTCTGG -3' and 5'-TATAGGATCCTCATTACTGGTTAATTTTAGG -3' were added and the PCR reaction was continued for 25 more cycles to amplify the complete gene. The modified *bin*B gene with two point mutations for two substitutions (His109Pro and Pro274Ser) was isolated and purified using gel purification kit (Qiagen). The point mutations were confirmed by nucleotide sequencing. The modified *bin*B gene was then cloned into *p*ET28a(+) expression vector using NdeI (NEB) and BamHI (NEB) restriction sites and T4 DNA ligase (NEB) and the construct was transformed into *E. coli* XL-10 gold competent cells under kanamycin (50 µg/ml) selection. Positively transformed colonies were selected through colony PCR using Taq DNA polymerase (Invitrogen). The modified *p*ET28a(+)-*bin*B construct was isolated

using plasmid purification kit (Qiagen) and transformed into BL21star(DE3) competent cells for protein expression.

S1.2. Expression and purification of rationally engineered BinB protein

For protein expression, overnight grown culture of *E. coli* harbouring *p*ET28a(+)-*bin*B construct was used to inoculate 1L LB medium supplemented with kanamycin (50 µg/ml) and grown further at 37 ºC at 150 rpm. At cell density of ~0.7 (OD_{600}), the temperature was lowered down to 18 ºC and protein expression was induced with 0.5 mM IPTG. Cells were grown further overnight at 18 ºC at 150 rpm. Later, cells were harvested at ~6000 rpm for 5 min and resuspended in lysis buffer (25 mM Tris, pH 8.0, 200 mM NaCl, 5 % glycerol, 2 mM DTT, 1 mM PMSF, 2mg/ml lysozyme) and kept on ice for 30 min. The cells were lysed by sonication at 30 % amplitude for 10 min (3 sec. ON, 5 sec. OFF) and centrifuged thereafter at 13,000 rpm for 30 min. to separate the soluble fraction and inclusion bodies. The soluble fraction was loaded onto Ni-IDA matrix pre-equilibrated with buffer A (25 mM Tris–HCl, pH 8.0, 200 mM NaCl, 25 mM Imidazole, pH 8.0). The poly-His tagged recombinant BinB protein was eluted over imidazole concentrations gradient (from 0–1 M) using buffer B (25 mM Tris–HCl, pH 8.0, 200 mM NaCl, 1 M Imidazole, pH 8.0) and adjudged on 10% SDS-PAGE gel for homogeneity.

S2. Expression and purification of deuterated BinB protein

To facilitate *E. coli* cell growth and dBinB protein expression in D_2O, a three-step adaptive approach was followed to adapt *E.coli* cells to D_2O based growth medium: LB in H_2O to LB in D_2O to M9+ in D_2O. A 5 ml LB/H_2O culture (contained in a 100 ml flask) was started from a fresh agar plate of *E. coli* BL21star (DE3) harbouring pET28a(+)-*bin*B construct. Cells were grown for 3 h at 37 °C at 150 rpm following which 500 µl from LB/H_2O culture were inoculated to 5 ml of fresh LB/D_2O medium (contained in a 100 ml flask). The cells were grown further at 37 °C for about 5 hr at 150 rpm till the cell density (OD_{600}) reached ~ 0.5 to 1. The overnight pre-culture was started by transferring the 5 ml of LB/D_2O culture into a 25 ml M9+/ D_2O medium (contained in a 250 ml flask). Cells were grown overnight at 37 °C at 150 rpm. The 25 ml pre-culture was then inoculated into 250 ml of M9+/ D_2O medium (contained in a 1L flask) and allowed to grow at 37 °C at 150 rpm till OD_{600} reached ~0.7. The shaker temperature was then reduced to 20 °C and protein expression was induced by adding 1mM IPTG. Cells were grown further for an extended period of 48 h at 20 °C before harvesting. Cells were harvested and lysed following the protocol similar for hydrogenous BinB protein (details in Supplementary Method S1.2) except that all the buffers used for deuterated BinB protein were made in 100% D_2O. The deuterated protein was purified to homogeneity using immobilized metal ion affinity chromatography.

Table S1 Composition of the M9+ medium used for the expression of deuterated BinB protein.

K_2HPO_4	19.0 g
KH_2PO_4	5.0 g
Na_2HPO_4	9.0 g
K_2SO_4	2.4 g
D-Glucose*	18 g
NH_4Cl*	5.0 g
Trace elements#	1.0 ml
MEM Vitamin solution (100X)	10.0 ml
$MgCl_2$	0.95 g

* D-Glucose and NH_4Cl were used as the sole carbon and nitrogen source, respectively.

# The trace element solution (as described by Cai et al., 2016) comprises the following per 100 ml: 0.6 g FeSO4 (7H2O), 0.6 g CaCl2 (2H2O), 0.12 g MnCl2 (4H2O), 0.08 g CoCl2 (6H2O), 0.07 g ZnSO4 (7H2O), 0.03 g CuCl2 (2H2O), 2 mg H3BO4, 0.025 g (NH4)6Mo7O24 (4H2O), 0.5 g ethylenediaminetetraacetic acid (EDTA).

Table S2 Determining the extent of deuteration from experimental SANS data.

Protein	Molecular Weight (kD)	Density (gm/cc)	Concentration (mg/ml)	$d\Sigma/d\Omega(Q=0)$ (cm^{-1})	Calculated SLD (cm^{-2})	Experimental SLD (cm^{-2})	Deuteration Z (%)
BinB	53.6	1.36	3.33	0.1372	1.90×10^{10}	3.47×10^{10}	16.2
dBinB	57.3	1.45	6.0	Contrast matched (~0)	7.77×10^{10}	6.40×10^{10}	76.7
Cqm1	64.6	1.36	3.33	0.2862	1.97×10^{10}	3.68×10^{10}	19.8

<u>Detailed mathematical calculation is as follows:</u>

Chemical Structure

BinB: C(2396) H(3690) N(646) O(725) S(14)

Cqm1: C(2894) H(4318) N(786) O(869) S(20)

Neuron Scattering Length (SL)

BinB: [2396*(0.6646) +3690*(-0.3741) +646*(0.9360) +725*(0.5803) +14*(0.2847)] $*10^{-12}$ cm

$=1.2413*10^{-9}$ cm

dBinB: [2396*(0.6646) +3690*(0.6671) +646*(0.9360) +725*(0.5803) +14*(0.2847)] $*10^{-12}$ cm

$=5.0833*10^{-9}$ cm

Cqm1: [2894*(0.6646) +4318*(-0.3741) +786*(0.9360) +869*(0.5803) +20*(0.2847)] $*10^{-12}$ cm

$=1.5537*10^{-9}$ cm

Protein Volume (V)

V=Mol Wt/(N_A*d)

BinB and dBinB: 6.5435*10^{-20} cm^3

Cqm1: 7.8864*10^{-20} cm^3

H_2O and D_2O: 30*10^{-24} cm3

Volume of hydrated water with Bin B= 462 *30 * 10^{-24} =1.3860*10^{-20} cm^3=17.5 % volume

Volume of hydrated water with Cqm1= 560 * 30 * 10^{-24} =1.6800*10^{-20} cm^3=17.5 % volume

Calculated Neutron Scattering Length Density (SLD)

SLD=SL/V

BinB: 1.90*10^{10} cm^{-2}

dBinB: 7.77*10^{10} cm^{-2}

Cqm1: 1.97*10^{10} cm^{-2}

D_2O: 6.40*10^{10} cm^{-2}

H_2O: -0.56*10^{10} cm^{-2}

Experimental Neutron Scattering Length Density (SLD) by SANS

BinB: 3.47*10^{10} cm^{-2}

dBinB: 6.40*10^{10} cm^{-2}

Cqm1: 3.68*10^{10} cm^{-2}

Calculation of Deuteration

BinB: 0.825* (1.90*(1-Z) +7.77*Z) +0.175*6.40=3.47 * Z=0.162=16.2%

dBinB: 0.825* (1.90*(1-Z) +7.77*Z) +0.175*6.40=6.40 * Z=0.767=76.7%

Cqm1: 0.825* (1.97*(1-Z) +7.67*Z) +0.175*6.40=3.68 * Z=0.198=19.8%

A

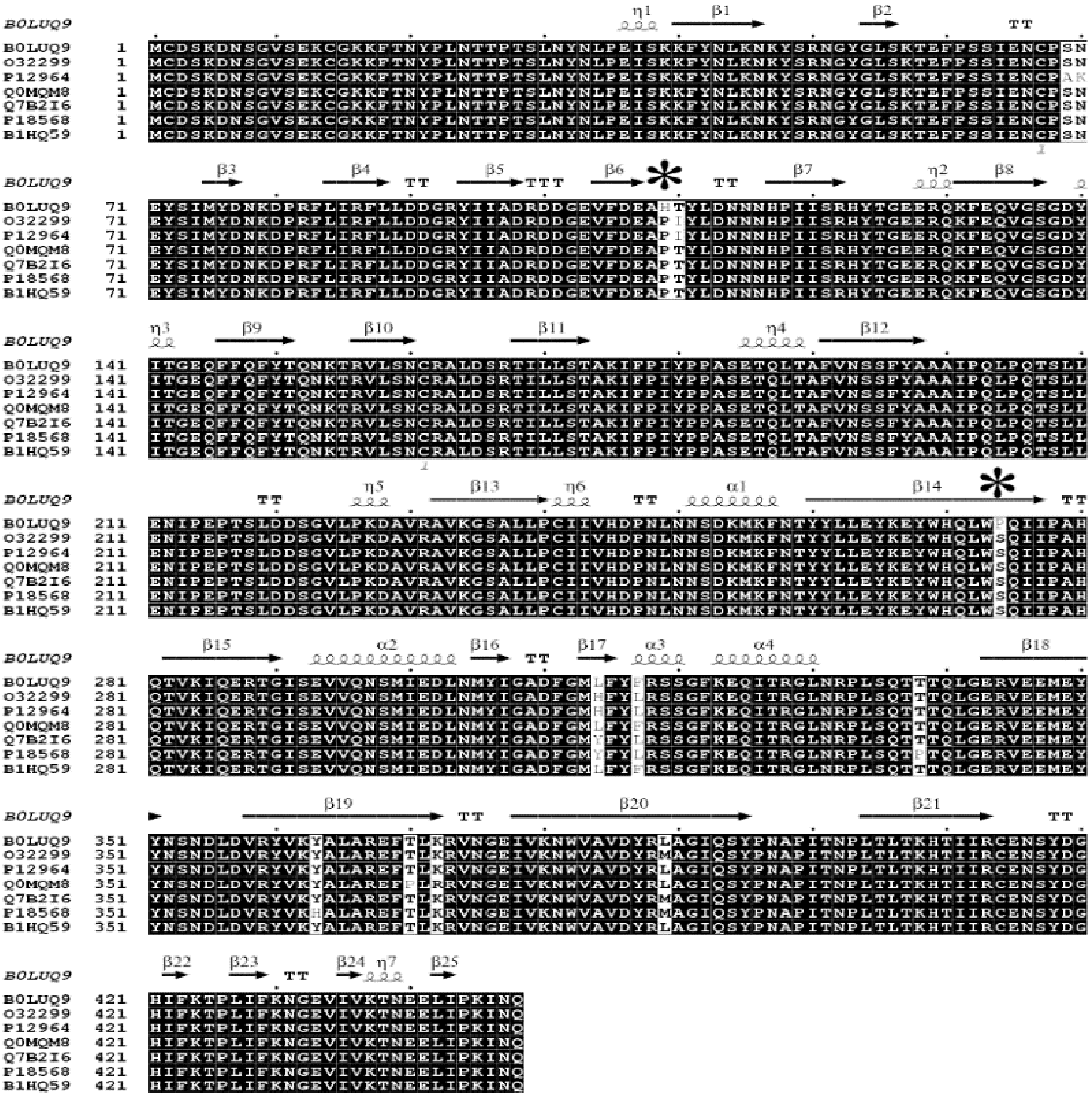
B0LUQ9
O32299
P12964
Q0MQM8
Q7B2I6
P18568
B1HQ59
η1 β1 β2 TT
β3 β4 TT β5 TTT β6 TT β7 η2 β8
η3 β9 β10 β11 η4 β12
TT η5 β13 η6 TT α1 β14 TT
β15 α2 β16 TT β17 α3 α4 β18
β19 TT β20 β21 TT
β22 β23 TT β24 η7 β25

B

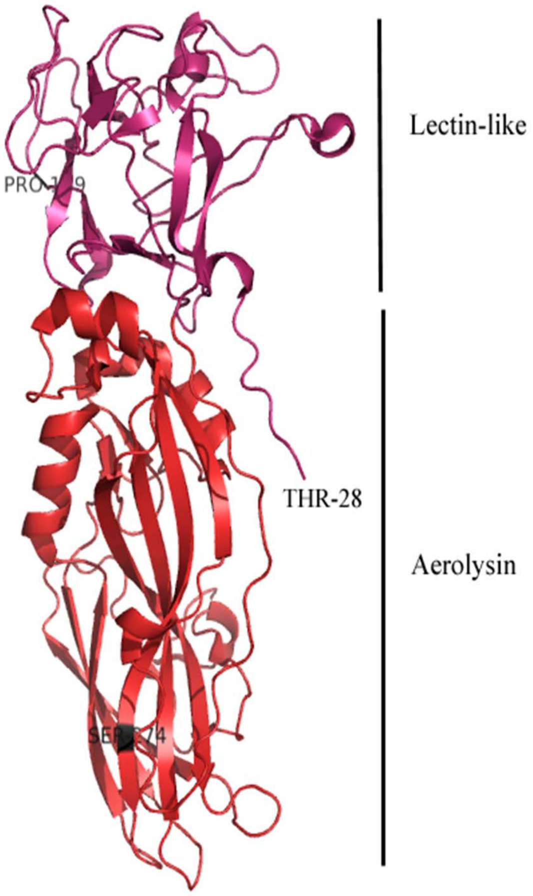

Figure S1 Rational engineering of BinB protein for enhancing protein solubility. **A)** Sequence alignment of all the different BinB protein sequences (*Ls*) available in the database. Alignment was made using Clustal Omega (Goujon *et al.*, 2010). Residues Marked with star, H109P and P274S, are varying only in the ISPC-8 sequence and conserved otherwise. Figure was prepared using EsPript (Robert & Gouet, 2014). **B)** Ribbon model for BinB crystal structure generated using Chimera (Pettersen *et al.*, 2004) depicting the location of residues, Pro-109 and Ser-274, and two known domains (lectin-like and aerolysin). Atomic coordinates for N-terminus 45 residues (including poly-His tag of 18 residues) are not available, which may constitute 3rd domain observed in the *ab initio* shape models of hBinB and Cqm1-hBinB generated by DAMMIN calculations.

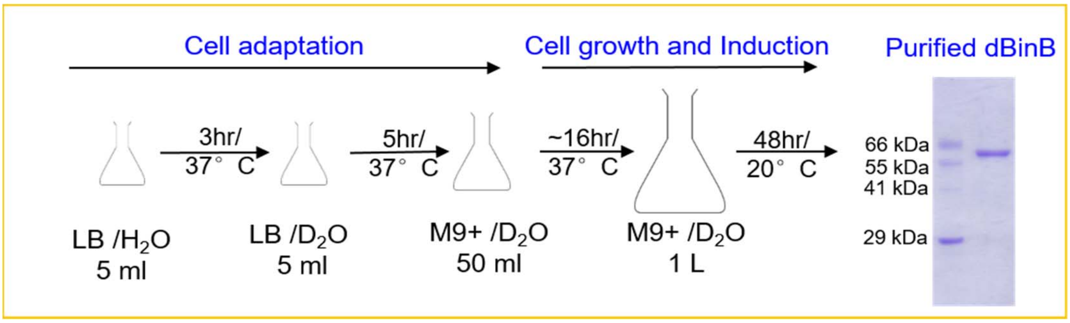

Figure S2 Schematic for the high yield expression protocol for deuterated protein using bacterial expression system and unlabelled carbon source in M9+/D_2O culture medium. The inset shows purified dBinB protein.

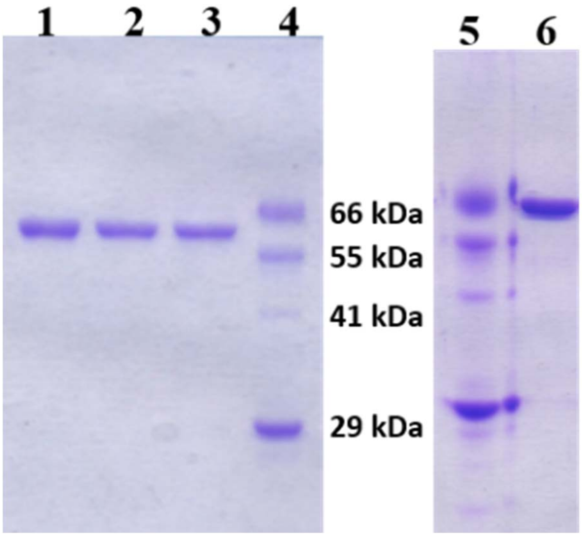

Figure S3 Electrophoretic analysis of the purified BinB and Cqm1 proteins (hydrogenous and deuterated) used for SANS study: 10% SDS-PAGE gel; Lane 1, BinB (in H_2O based buffer); Lane 2, BinB (in D_2O based buffer); Lane 3, dBinB; Lane 4 & 5, protein molecular weight markers; lane 6, Cqm1 (in D_2O based buffer).

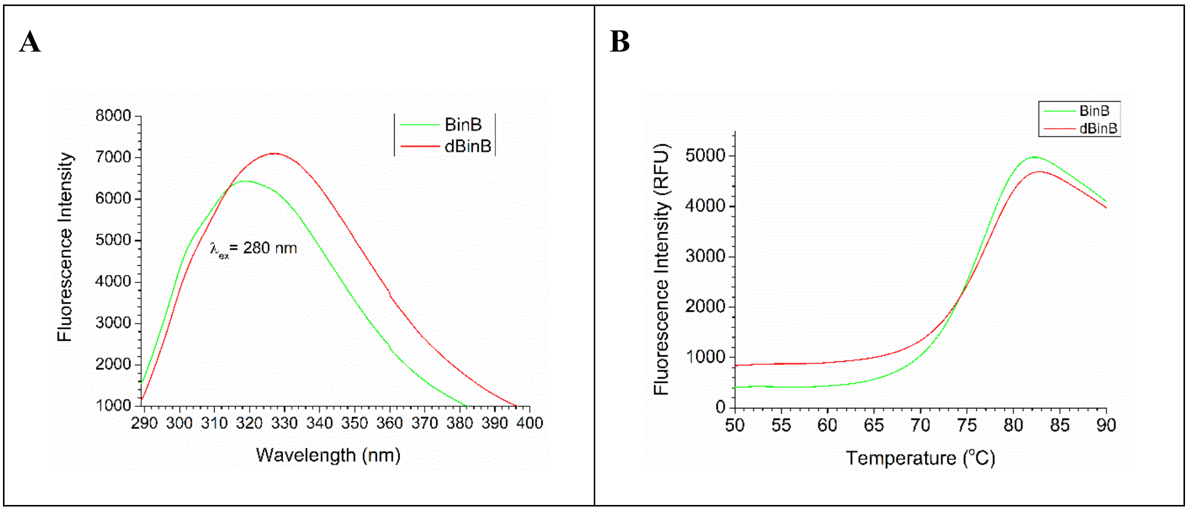

Figure S4 Characterization of purified dBinB. A) Intrinsic (trp) fluorescence spectra showing a properly folded dBinB (λmax, 327 nm) protein similar to BinB (λmax, 317 nm). B) Thermal melting profile using differential scanning fluorimetry (DSF) reveals that dBinB displays thermal stability similar to hydrogenous BinB protein.

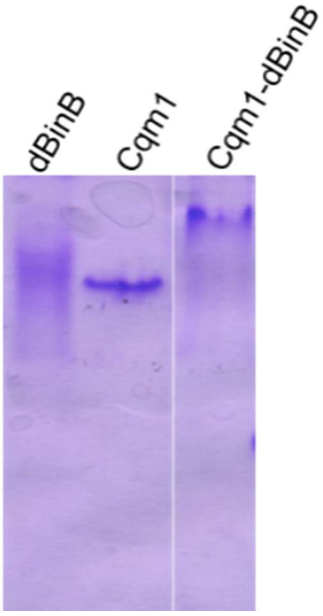

Figure S5 Electrophoretic characterization of the Cqm1-BinB complex formation. 10% Native-PAGE shows that Cqm1exhibits retarded mobility in the presence of BinB, suggesting that Cqm1 and BinB proteins form stable heteromeric complex.

A

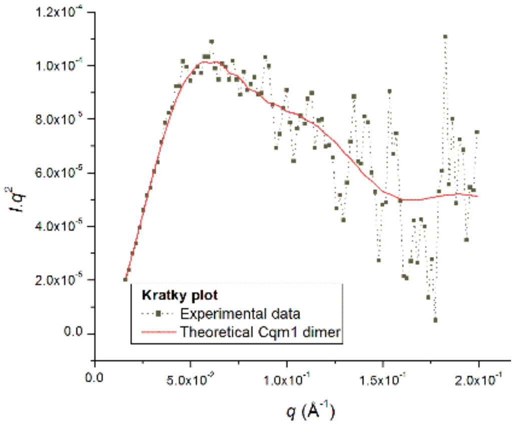

B

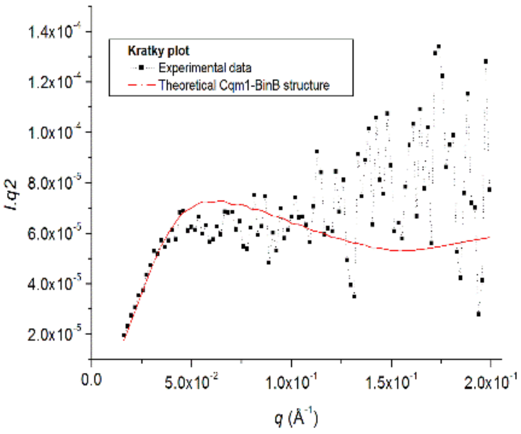

C

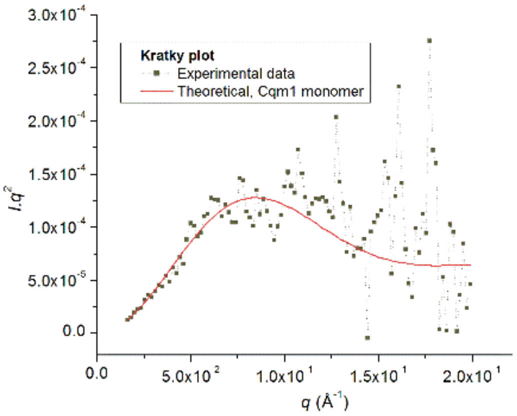

Figure S6 Kratky plots. (A) Cqm1 dimer in solution, (**B**) Cqm1-hBinB complex and (**C**) Cqm1-dBinB complex. The points (and back dashed lines) are experimental SANS data. Theoretical plot (red line) is based on fitted Intensity values from the structural models. For Cqm1-dBinB, the theoretical plot is based on estimated values for Cqm1 monomer as scattering due to “contrast matched” dBinB is observed to be eliminated.

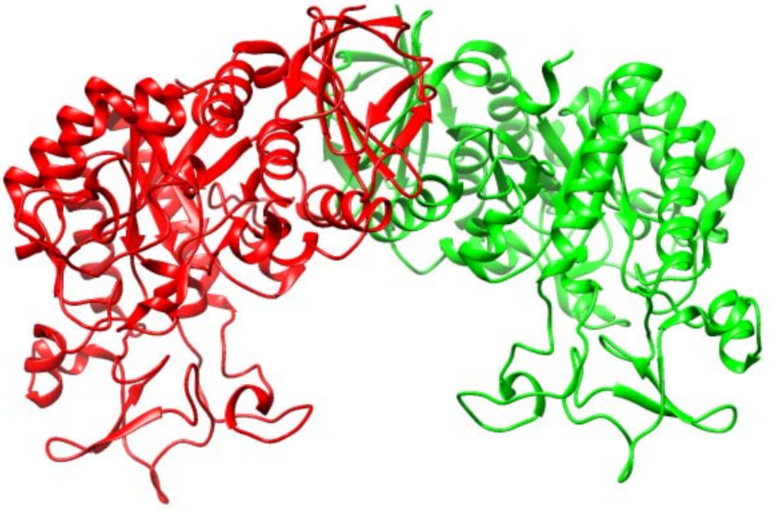

Ribbon model for Cqm1 dimer crystal structure (PDB: 6K5P). Cqm1 crystal structure depicts weak biological dimer with the two monomers (A, red; B, green) held together by solvation free energy gain ~ 4.4 kcal/mole. The ribbon model was generated using Chimera (Pettersen *et al.*, 2004).
